# Relapsed angioimmunoblastic T-cell lymphoma presenting as psoriasiform dermatitis

**DOI:** 10.1016/j.jdcr.2023.01.012

**Published:** 2023-01-28

**Authors:** Spencer McClure, Brooks Kimmis, Haleigh Harper, Maryam Abdo, Edward Seger, Ting Wang

**Affiliations:** a Division of Dermatology, Department of Internal Medicine, University of Kansas Medical Center, Kansas City, Kansas; b University of Kansas School of Medicine, Kansas City, Kansas; c Department of Pathology, University of Kansas Medical Center, Kansas City, Kansas

**Keywords:** angioimmunoblastic T-cell lymphoma, dermatology, Follicular Helper TCell, oncology, psoriasiform dermatitis, AITL, angioimmunoblastic T-cell lymphoma, CD, cluster of differentiation

## Introduction

Angioimmunoblastic T-cell lymphoma (AITL) is a subtype of peripheral T-cell lymphoma that makes up 1% to 2% of all non-Hodgkin lymphoma. AITL’s cell of origin is the follicular T-helper cell (CD [cluster of differentiation]10, BCL6, PD1, CXCL13, and ICOS positive), which normally functions as an important checkpoint in the process of B-cell activation and differentiation. This condition is difficult to diagnose due to high clinical variability, likely leading to late recognition and poor outcomes.^1^ Our case report reviews yet another unique skin manifestation of AITL; one mimicking psoriasiform dermatitis with intermittent trailing scale.

## Case presentation

A 72-year-old male with past medical history of systemic AITL was admitted due to worsening dyspnea and a new rash. The patient had previously completed chemotherapy, including 6 cycles of the “A+CHP” regimen (brentuximab vedotin, cyclophosphamide, doxorubicin, and prednisone) followed by 2 cycles of “ICE” (ifosfamide, carboplatin, and etoposide) therapy. The patient had completed this chemotherapy regimen about 1 week prior to the development of skin changes. When describing the rash, the patient noted extreme pruritus over the preceding 2 weeks, especially over his scapula and back. The patient reported some symptomatic relief from topical betamethasone, but the benefits were only fleeting. He denied any pain, oral involvement, fevers, chills, or recent weight loss.

The patient’s complete blood count was remarkable for a white blood cell count of 18.2 K/uL with a 49% lymphocytic predominance, as well as a hemoglobin count of 8.8 g/dL. His comprehensive metabolic panel was unremarkable. A respiratory viral panel was negative, including a negative COVID-19 polymerase chain reaction. Blood cultures showed no growth.

**Fig 1:**
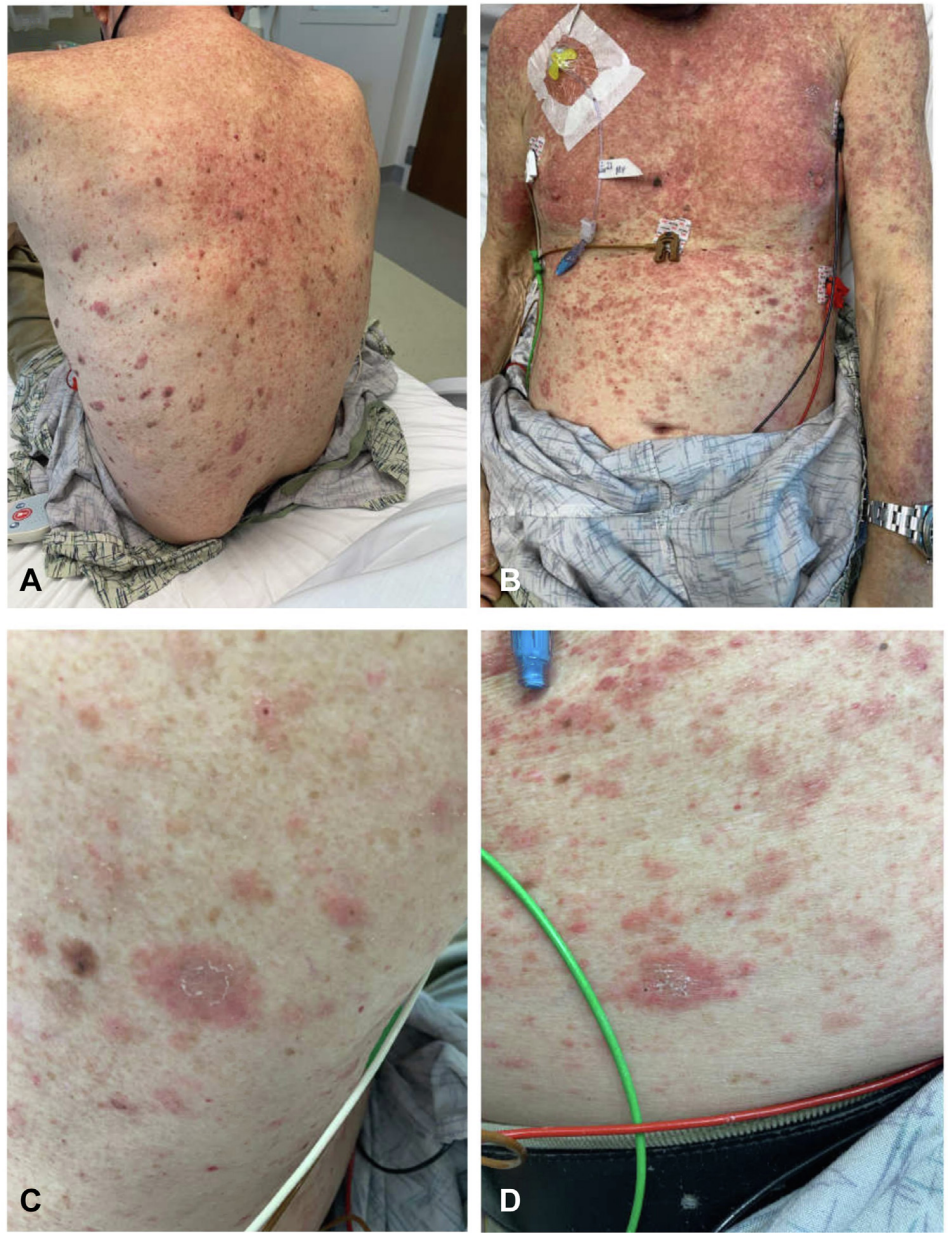
**A,** Red-pink macules and thin papules coalescing into thin plaques and patches on the back **(B)** and chest. **C** and **D,** Thin plaques with trailing scale on the patient's back.

Dermatology was consulted due to the patient’s new onset skin rash. The patient’s physical exam was significant for red-pink macules and thin papules coalescing into thin plaques and patches predominantly on his chest as well as his arms, abdomen, and back with few on his lower extremities. Some lesions had trailing scale. Islands of sparing were present. Additionally, there was a diffuse red patch on his face and frontal scalp with some scale (Fig 1).

Given the exam findings of plaques with intermittent trailing scale and recent chemotherapy initiation, diagnoses of morbiliform drug eruption, pityriasis rubra pilaris, psoriasis, and pityriasis rosea-like drug eruption were considered. A punch biopsy was performed on the patient’s right abdomen. The dermatology team recommended discontinuing all nonessential medications, continuing the prednisone taper previously initiated by the patient’s primary care team, and applying clobetasol 0.05% cream to the affected areas of the body as well as hydrocortisone 2.5% cream to the face.

**Fig 2:**
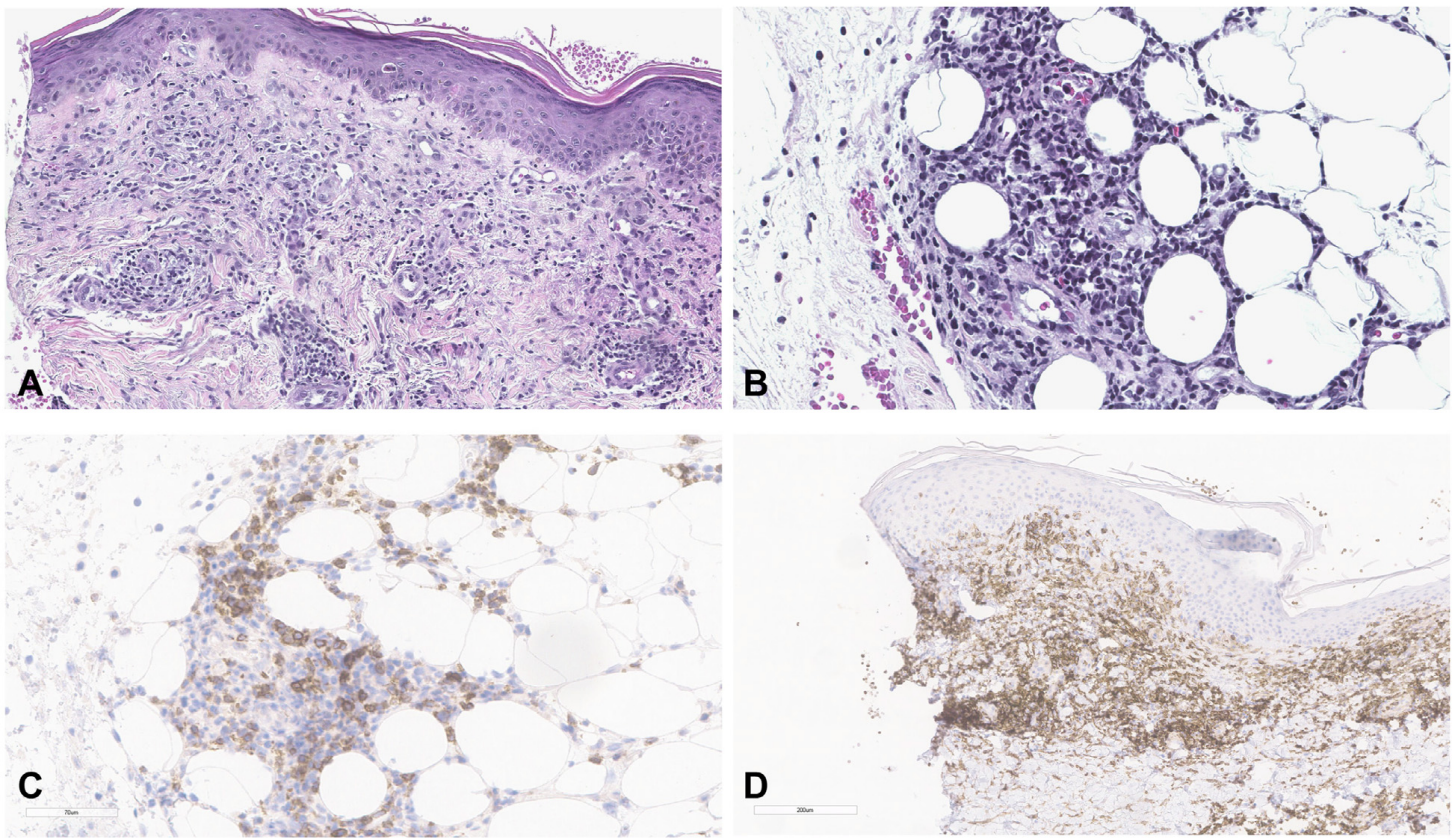
**A,** Perivascular lymphoid infiltrate in the superficial dermis **(B)** Lymphocytic involvement of the subcutaneous fat **(C)** PD-L1 immunostain positivity **(D)** and CD4 immunostain positivity.

The patient’s histopathology was significant for a superficial and deep infiltrate of small lymphocytes and scattered eosinophils in the perivascular adventitial dermis and focally in the subcutis in a lobular distribution. Immunohistochemical staining demonstrated T lymphocytes that expressed the following markers: CD3, CD4, CD5, CD7, CD8, PD1, BCL6, and CD10. CD20 was negative. Given that the AITL cell of origin is the follicular T-helper cell, the positive staining supports the diagnosis of cutaneous AITL, rather than the psoriasiform differential as was suspected clinically. At his next follow-up visit, the patient’s oncology team noted the patient’s skin lesions and new onset diffuse bone pain as clinical progression of AITL leading to initiation of romidepsin and duvelisib. Over the month-long course of the first treatment cycle, the patient showed near resolution of his skin lesions and previously noted bone pain. Due to this clinically significant improvement, the patient’s oncology team opted to continue the treatment regimen (Fig 2).

## Discussion

Overall, AITL makes up approximately 1% to 2% of all non-Hodgkin lymphoma diagnoses per year and shows a low incidence in the United States, representing only 0.05 new cases per 100,000 people.^2^^,^^3^ AITL has a predisposition for an older population, with a median patient age of 65 years at diagnosis, but no predilection based on sex has been identified in the literature thus far.^4^^,^^5^ AITL has been well-described as a disease accompanied by highly variable clinical presentations, with signs and symptoms including hepatosplenomegaly, ascites, gastrointestinal abnormalities, myalgias, cough, and peripheral neuropathy. However, 2 of the most reported findings remain constitutional symptoms (night sweats, fevers, and chills), and skin involvement, which can be seen in up to 50% of patients.^6^ Unfortunately, consistent recognition and diagnosis remains difficult based on cutaneous findings, as the reported skin manifestations associated with AITL are highly variable. Skin changes have been described nonspecifically as maculopapular, morbilliform, erythrodermic, nodular, and urticarial. Pruritus is also reported in over 50% of patients with skin involvement.^7^ Histopathologic findings most commonly consist of vasculitis, perivascular infiltrate, and vascular hyperplasia. Immunohistochemistry often plays a significant role in making a diagnosis of AITL. The cells are commonly positive for multiple antigens representative of T-follicular helper cells such as CD10, PD1, BCL6, PD1, CXCL13, ICOS, CXCR5, and others. However, it is important to note that these markers are not exclusive to AITL and can also be found in other cutaneous T-cell lymphomas, and primary cutaneous CD4+ T-cell lymphoproliferative disorder.^8^ Due to the lack of explicit diagnostic findings from skin biopsy, diagnosis of AITL is often missed or delayed until lymphadenopathy leads to eventual lymph node biopsy and subsequent diagnosis.^9^ Thus, AITL is overwhelmingly diagnosed in late stages, likely due in part to the nonspecific clinical and histological findings outlined above. Prognosis is predictably poor, with a 5-year overall survival rate of just 26% to 36%.^10^

In summary, we present a case of AITL with unique skin presentation that mimics psoriasiform dermatitis with intermittent trailing scale. Our case highlights the importance of considering the diagnosis of cutaneous AITL in dermatoses that are atypical or refractory to treatment.

## Conflicts of interest

None disclosed.
